# Physicians’ Considerations and Practice Recommendations Regarding the Use of Sodium-Glucose Cotransporter-2 Inhibitors

**DOI:** 10.3390/jcm11206051

**Published:** 2022-10-13

**Authors:** Serge A. Jabbour, Nasrien E. Ibrahim, Christos P. Argyropoulos

**Affiliations:** 1 Division of Endocrinology, Diabetes & Metabolic Diseases, Sidney Kimmel Medical College, Thomas Jefferson University, 211 S Ninth Street, Suite 600, Philadelphia, PA 19107, USA; 2 INOVA Heart and Vascular Institute, Falls Church, VA 22042, USA; 3 School of Medicine, University of New Mexico, Albuquerque, NM 87131, USA

**Keywords:** sodium-glucose cotransporter-2 inhibitors, cardiovascular, renal, safety, type 2 diabetes

## Abstract

Sodium-glucose cotransporter-2 inhibitors (SGLT-2is) (canagliflozin, dapagliflozin, empagliflozin, and ertugliflozin), although initially developed as glucose-lowering drugs, provide significant beneficial effects on cardiorenal outcomes, including heart failure, regardless of type 2 diabetes status. Integration of SGLT-2is into clinical practice requires practical guidance for physicians about their use. To overcome physicians’ clinical inertia for SGLT-2i use, including addressing safety, potentially a barrier to their use, a roundtable discussion with physicians from three specialties (cardiology, endocrinology, and nephrology) was conducted. This review summarizes the physicians’ clinical experience and recommendations about SGLT-2i use across different patient populations, taking into consideration the beneficial effects of SGLT-2is and their safety. The key aspects discussed regarding SGLT-2i safety include acute effects on kidney function (estimated glomerular filtration rate acute dip upon SGLT-2i initiation and acute kidney injury), volume depletion, diabetic ketoacidosis, genitourinary infections, hyperkalemia, and hypoglycemia. To mitigate any potential risks related to SGLT-2i safety, physicians can make minor adjustments to an individual patient’s treatment plan, while retaining the SGLT-2i cardiorenal benefits for effective disease management. Recognition by physicians that the benefits of SGLT-2i use on clinical outcomes outweigh the risks will result in the integration of SGLT-2is into clinical practice and lead to improved patient care and outcomes.

## 1. Introduction

There has been an increased awareness among physicians about the beneficial effects of sodium-glucose cotransporter-2 inhibitors (SGLT-2is) [1], originally developed as glucose-lowering agents, on cardiovascular (CV) and renal outcomes (independent of their glycemic effects), which has been driven in part by clinical trial evidence and practice guideline updates [2,3]. Consequently, it is important that physicians are aware of SGLT-2is’ mechanism of action and their potential to improve patients’ outcomes, while considering the potential for adverse effects, and of how to best integrate SGLT-2is into their clinical practice, as a means to better address the individual patient’s therapeutic goals. This article provides an overview of the CV and renal benefits of SGLT-2is in patients with and without type 2 diabetes (T2D) and addresses the potential physician-related barriers to the effective use of these drugs in routine clinical practice, including aspects related to safety that may contribute to clinical inertia.

## 2. SGLT-2is

**Figure 1:**
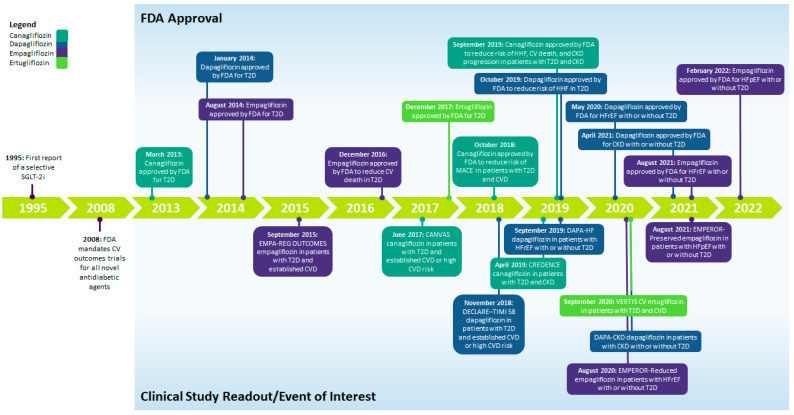
Timeline summarizing the development and approval of SGLT-2is. CKD, chronic kidney disease; CV, cardiovascular; CVD, cardiovascular disease; FDA, US Food and Drug Administration; HF, heart failure; HFpEF, heart failure with preserved ejection fraction; HFrEF, heart failure with reduced ejection fraction; HHF, hospitalization for heart failure; SGLT-2i, sodium-glucose cotransporter-2 inhibitor; T2D, type 2 diabetes.

The first SGLT-2i, phlorizin, a plant-based glycoside of the flavonoid group, was discovered 150 years ago. Phlorizin was not a viable therapeutic agent, because it had poor oral absorption, lacked specificity, and had severe side effects in animal models [4,5,6]. Selective SGLT-2is were first reported in the 1990s, followed by the development of canagliflozin, dapagliflozin, empagliflozin, and ertugliflozin [6]. In 2008, the US Food and Drug Administration (FDA) mandated that CV outcome trials (CVOTs) for novel antidiabetic agents be conducted [7] to show that glucose-lowering agents do not carry excess CV risk. Consequently, CVOTs in patients with T2D and established atherosclerotic CV disease (ASCVD) or high CV risk (EMPA-REG OUTCOME, CANVAS, DECLARE–TIMI 58, and VERTIS CV) were conducted for several SGLT-2is [8,9,10,11]. Figure 1 shows SGLT-2i development leading up to approvals for different indications. The chemical structures of the four SGLT-2is with US FDA approval are presented in Supplementary Figure S1. 

**Figure 2:**
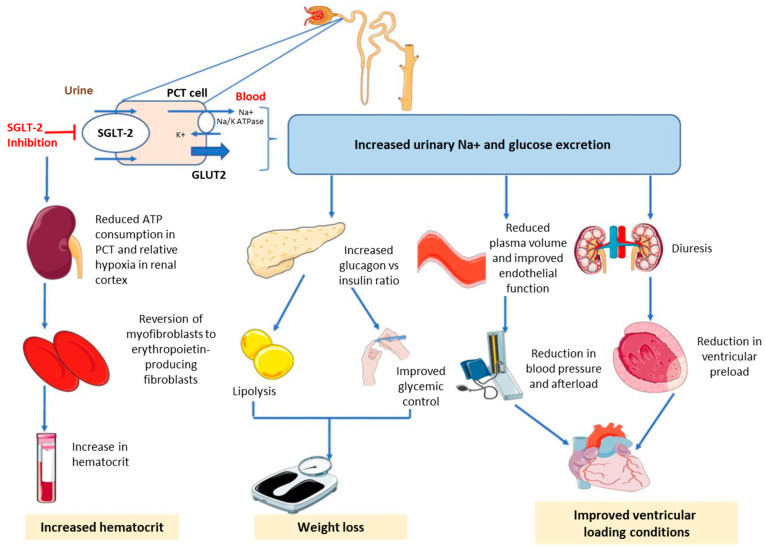
Mechanisms of action of sodium-glucose cotransporter-2 inhibitors. ATP, adenosine triphosphate; GLUT, glucose transporter; K+, potassium; Na+, sodium; PCT, proximal convoluted tubule; SGLT-2, sodium-glucose cotransporter-2. ©2020 S. Joshi. [19] Re-use permitted under CC BY 4.0. Published by *BMJ*. The original figure published in Joshi et al., was created using Servier Medical Art (http://smart.servier.com).

SGLT-2is act by blocking the paired reuptake of sodium and glucose in the renal proximal tubule, thereby promoting urinary glucose and sodium excretion [2]. Reduction in glucose reabsorption in the kidney and lowering of plasma glucose are independent of insulin levels or peripheral insulin resistance [5,12]. Apart from glycemic control, the beneficial effects of SGLT-2is encompass weight loss (glucosuria-induced energy loss) and small blood pressure (BP) reduction (due to osmotic diuresis and intravascular volume contraction) [13]. Delivery of sodium to the distal tubule increases tubuloglomerular feedback and reduces intraglomerular pressure [14,15,16,17]. Combined with osmotic diuresis, this leads to a reduction in volume overload, reduced blood pressure, and lower preload and afterload, which may preserve renal function and have beneficial effects on cardiac remodeling [14,15,18]. Figure 2 shows the mechanisms of action for SGLT-2is.

Although designed to lower blood glucose, SGLT-2is demonstrated broad CV and renal benefits in CVOTs [20]; that is, SGLT-2is were found to reduce the risk of major adverse CV events in patients with T2D who had established ASCVD or high CV risk and also the risk of hospitalizations for heart failure (HF) and CV death [8,9,10,11]. Moreover, a reduction in the risk of chronic kidney disease (CKD) progression was observed with SGLT-2i therapy in patients with or without T2D, resulting in a lower risk of the composite end point of sustained decline in estimated glomerular filtration rate (eGFR) of at least 50%, end-stage renal disease, and death due to renal causes [21,22]. Table 1 summarizes the major outcome trials demonstrating the glycemic, CV, and renal effects of SGLT-2is [8,9,10,11,21,22,23,24,25,26].

The clinical evidence from these outcome trials, demonstrating the cardio- and renoprotective benefits of SGLT-2is in patients with or without T2D, has led to the expansion of the FDA-approved indications (ASCVD, CKD, CV disease (CVD), and/or HF with reduced ejection fraction (HFrEF) or with preserved ejection fraction (HFpEF)) for SGLT-2is (Figure 1) [27,28,29]. Clinical practice guidelines have also been updated to include SGLT-2i therapy for the prevention of CV and renal complications (Table 2) [30,31,32,33,34,35,36,37].

For optimal prescribing of SGLT-2is, a complete understanding of the potential risks associated with their use, in addition to their benefits, is required. Table 3 summarizes the safety profiles of SGLT-2is in patients with T2D and established CVD or high CVD risk, with T2D and CKD, with HF with or without T2D, and with CKD with or without T2D [8,9,10,11,21,22,23,24,25,26]. Among physicians, the key safety considerations that may prevent prescription of SGLT-2is are volume depletion and associated acute kidney injury (AKI), hypoglycemia, diabetic ketoacidosis (DKA), and genitourinary infections [27,28,29,38]; however, adverse events with SGLT-2is are generally manageable, and serious adverse events are rare [12]. Adverse effects can be mitigated by making minor adjustments tailored to meet specific patient requirements for effective disease management and/or to address intermittent illness or major surgery (“sick-day” strategy) [39].

## 3. Overcoming Physician-Related Barriers to SGLT-2i Use

**Figure 3:**
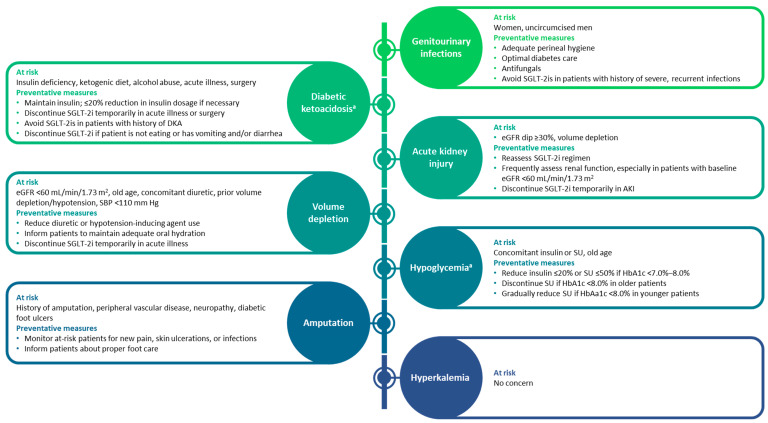
Adverse events associated with SGLT-2is and proposed preventative measures [36,37,40]. ^a^ Occurs only in patients with type 2 diabetes. AKI, acute kidney injury; DKA, diabetic ketoacidosis; eGFR, estimated glomerular filtration rate; HbA1c, glycated hemoglobin; SBP, systolic blood pressure; SGLT-2i, sodium-glucose cotransporter-2 inhibitor; SU, sulfonylurea.

Evidence from several clinical outcome trials has demonstrated the cardio- and renoprotective effects of SGLT-2is in patients with established CVD or at risk for CVD, including HF or CKD, with or without T2D; however, physicians’ practice and perceptions have impeded the use of SGLT-2is [3]. To overcome clinical inertia regarding the use of SGLT-2is in routine clinical practice and to address any key safety aspects associated with SGLT-2is, a roundtable discussion including physicians from three different specialties (cardiology, endocrinology, and nephrology) was conducted. This section considers the opinions of these physicians on key aspects related to the use of SGLT-2is and their clinical experience across differing patient populations. Their varied treatment approaches are summarized in Figure 3.

### 3.1. Acute Effects on Renal Function upon SGLT-2i Initiation

#### 3.1.1. eGFR Acute Dip

An acute decrease in eGFR of ~2–5 mL/min/1.73 m^2^, also known as “eGFR dip”, can occur within the first 2–4 weeks of SGLT-2i treatment initiation [41,42,43], but it is typically followed by a partial recovery of kidney function by week 12 and then an attenuation of the slope of the decline in eGFR compared with that of placebo [42,44]. An acute eGFR dip is more likely in patients receiving thiazides and/or loop diuretics at baseline and those belonging to a higher Kidney Disease: Improving Global Outcomes risk category [42,43]. An initial >10% decrease in eGFR, which does not require that SGLT-2is be discontinued, has been reported in 25% of patients; eGFR decreases of >30% are rare [42,43], and, in these patients, SGLT2i therapy should be temporarily discontinued until eGFR returns to baseline [43]. In patients with lower eGFR (stage 4 CKD; eGFR <30 mL/min/1.73 m^2^), the eGFR dip is smaller than that observed in patients with stage 2/3 CKD (eGFR ≥30–≤90 mL/min/1.73 m^2^; 1.42 mL/min/1.73 m^2^ compared with 2.56 mL/min/1.73 m^2^, respectively, from baseline to 2 weeks); this indicates that the eGFR dip is attenuated in patients with stage 4 CKD [45]. In addition, the initial eGFR dip is not associated with reduced benefit with respect to cardiorenal outcomes [42,45].

On the basis of data from the EMPA-REG OUTCOME [11], DAPA-CKD [21], VERTIS CV [8], and CREDENCE [22] trials, as well as real-world evidence [46], increased frequency in monitoring, beyond that indicated by a patient’s diabetes and kidney disease, is not warranted unless there are signs and symptoms of volume depletion (orthostatic hypotension, BP <120/70 mmHg) in specific patients, such as those who are aged >65–70 years or those receiving high-dose diuretics [43].

#### 3.1.2. AKI

There may be a perception of an increased risk of AKI with the use of SGLT-2is. However, the incidence of AKI, defined as a serum creatinine increase of >0.3 mg/dL (within 48 h) or >1.5 times the baseline value (prior 7 days), is low with SGLT-2is, as reported in clinical and real-world studies [21,22,47,48,49]. Although there have been post-marketing reports of AKI with SGLT-2is in association with volume depletion [27,28,29,38], clinical trials showed a lower risk of AKI (vs. placebo) with dapagliflozin (serious adverse events, 1.8% vs. 2.4% of patients) in DAPA-CKD [21] and in DAPA-HF (serious adverse events, 1.0% vs. 1.9% of patients) [23] and with canagliflozin (adverse events, 16.9 vs. 20.0 events per 1000 patient-years) in CREDENCE [22]. Similarly, the rate of AKI was not increased compared with that of placebo for empagliflozin, canagliflozin, or dapagliflozin in EMPA-REG OUTCOME [24], CANVAS [9], and DECLARE–TIMI 58 [10], respectively. Furthermore, a meta-analysis of 18 trials (*n* = 156,690) showed that the risk of AKI with SGLT-2is was 24% lower than that with placebo [50]. An increased risk of AKI with SGLT-2is was not observed in an observational study of patients from two CKD registries [47] or in a retrospective study comparing AKI incidence with SGLT-2is and other glucose-lowering drugs [48]. In a meta-analysis of the CV, HF, and renal outcome trials of SGLT-2is compared with placebo, the risk for AKI was reduced by 25%, irrespective of the specific SGLT-2i used, the population included (patients with or without T2D), and the patients’ underlying kidney disease, diabetes, or HF status [1].

Although AKI rarely occurs with SGLT-2i use [48], renal function should be assessed prior to the initiation of the SGLT-2i and regularly afterward [36,37,40]. Patients with an eGFR of <60 mL/min/1.73 m^2^ at initiation may require more frequent assessment of renal function because of their underlying CKD rather than their use of an SGLT-2i. In patients with AKI, SGLT-2i treatment should temporarily be discontinued until renal function improves [35,36,37,46].

### 3.2. Volume Depletion

SGLT-2is cause glucosuria-induced osmotic diuresis and natriuresis, which can result in a total fluid loss of ~1–2 kg and sodium loss of 6% in the first 1–2 weeks of treatment, which subsequently stabilizes [51]. This plasma volume reduction leads to a sustained lowering of BP by 4–6 mmHg (systolic)/1–2 mmHg (diastolic) [12]. Patients at increased risk of volume depletion are those with eGFR <60 mL/min/1.73 m^2^, those with older age (≥ 65 years old), and those receiving loop diuretics [28,29]. In general, SGLT-2is are not hypotensive agents, as the reductions in systolic BP are small in magnitude. However, an increased risk of volume-depletion-related adverse effects was reported in patients with HFrEF and baseline systolic BP of <110 mmHg in the DAPA-HF trial [52].

Prior to SGLT-2i initiation, volume depletion can be addressed by a reduction in the dose of diuretics (based on the individual patient’s volume status) or hypotension-inducing agents (angiotensin-converting enzyme inhibitor and angiotensin II receptor blocker) [12,39,53]. Although SGLT-2is do not appear to increase the risk of orthostatic hypotension in randomized controlled trials [54], volume correction is necessary in patients with volume depletion or existing hypotension to avoid potential orthostatic hypotension upon initiation of SGLT-2i treatment [39]. After SGLT-2i initiation, close monitoring of body weight, BP, and volume status is recommended, and patients should be counseled about avoiding dehydration and the risk of orthostatic hypotension, particularly in the first week of therapy [12,40,55]. In the event of an acute illness, major surgery, or ingestion of nothing or a marked reduction in oral intake, SGLT-2i treatment should be temporarily discontinued until recovery, following the “sick-day” strategy [39,55].

### 3.3. DKA

Patients with a lack of endogenous insulin (certain insulin-dependent T2D or type 1 diabetes) are at increased risk of DKA, with a higher incidence observed in patients with type 1 diabetes [39]. An increased risk of non-SGLT-2i-associated DKA has been attributed to precipitating events including stressful events (i.e., infections, alcohol abuse, surgery, stroke, myocardial infarction, or trauma), because they increase the production of counter-regulatory stress hormones such as glucagon, leading to lipolysis and promotion of ketone formation in the presence of insulin deficiency [56,57]. Although DKA associated with SGLT-2i use is rare (~0.5 per 1000 patient-years) [12], a meta-analysis indicated that the risk of DKA may be increased with SGLT-2is compared with that with placebo [58]. DKA associated with SGLT-2i use may be caused by underlying insulin insufficiency, an increased rate of fatty acid oxidation, reduced ketone clearance, or stimulation of glucagon secretion [59].

The usual symptoms of DKA are nausea, vomiting, malaise, abdominal pain, and fruity odor on the breath [12,39]. In patients receiving SGLT-2is, the diagnosis of DKA can be delayed because blood glucose may be normal or only slightly elevated (i.e., euglycemic DKA), as opposed to classic DKA in which the patients have hyperglycemia [56]. Therefore, assessment of serum ketones or urine ketones is needed in patients receiving SGLT-2is who present with DKA symptoms and slightly elevated blood glucose [60]. DKA is usually treated with insulin and fluid and electrolyte replacement [39]. When initiating SGLT-2i treatment in a patient receiving insulin and considering a reduction in insulin dosage, initial reductions of >20% should be avoided to prevent triggering DKA [40]. In patients with acute illness or 3 days before a major surgery, the “sick-day” strategy should be considered, whereby SGLT-2i treatment is temporarily discontinued to minimize the risk of DKA [36,40]. SGLT-2i therapy should also be stopped in patients who are not following a proper diet and/or in those who are inadequately hydrated. Moreover, factors in the patient history that may predispose to ketoacidosis should be considered before starting SGLT-2i therapy [2,12,39]. There is no evidence of ketoacidosis with SGLT-2is in patients without T2D [21,23]. Therefore, the physicians agreed that, if indicated, SGLT-2is can be prescribed without hesitation to patients with HF or CKD who do not have T2D.

### 3.4. Genital Mycotic Infections, Urinary Tract Infections, and Fournier’s Gangrene

Genital mycotic infections (GMIs) are primarily fungal infections of the urogenital area (e.g., candidiasis), whereas the urinary tract infections (UTIs) in patients receiving SGLT-2is are primarily bladder infections [61]. SGLT-2i-induced glucosuria increases the risk of GMIs and UTIs, with GMIs occurring at a higher rate than UTIs; these infections are common in women with a history of genital infection and uncircumcised men (albeit comparatively less frequent) [12,62]. A meta-analysis of clinical studies showed increased odds of GMIs with SGLT-2is vs. placebo (odds ratio (OR), 3.87 (95% CI, 3.18–4.71)), whereas the odds of UTIs were lower than for GMIs (OR, 1.08 (95% CI, 1.00–1.18)) [1].

GMIs usually occur soon after initiation of SGLT-2i treatment, especially in patients with T2D, and are typically mild in severity [62,63]. They can be managed effectively with antifungals (either topical or systemic) and by counseling patients about maintaining adequate personal hygiene to mitigate the risk of infections [63]. An ~10-fold reduction in the incidence of GMIs may be achieved by regular washing of the urogenital region, particularly after each void and before going to bed [64]. Furthermore, advising female patients to wear cotton underwear may reduce the risk of vaginal candidiasis [65]. However, SGLT-2is should probably be avoided in females with a history of severe, recurrent fungal infections or in patients with paraparesis, neurogenic bladder, or an indwelling urinary catheter [39,63]. In general, the physicians agreed that the benefits of SGLT-2is far outweigh the risks of infection and that they initiate SGLT-2i treatment even in patients with a history of GMIs or UTIs after a discussion with the patient.

Fournier’s gangrene is a rare serious infection of the genital and perigenital area (incidence rate, 0.61 cases per 1000 person-years), primarily attributed to poorly controlled diabetes [66,67]. Although cases of Fournier’s gangrene were reported with SGLT-2i therapy by the FDA in 2018 [68], they have been attributed to patient-specific risk factors including poorly controlled diabetes, advanced age, obesity, and alcohol abuse [39,67]. Physician education about the signs and symptoms of this condition and the importance of patient monitoring can result in early diagnosis to mitigate the outcome effectively [67].

### 3.5. Hyperkalemia

There may be a perception that SGLT-2is are associated with an increased risk of hyperkalemia, based on the findings of a pooled analysis of phase 3 studies, which showed elevated serum potassium with canagliflozin 300 mg in patients with moderate impairment of renal function [69]. However, the incidence of hyperkalemia was uncommon in the canagliflozin CANVAS program [70] and in the canagliflozin CREDENCE study [22]. Moreover, a meta-analysis indicated that canagliflozin, dapagliflozin, and empagliflozin (with or without concomitant use of mineralocorticoid receptor antagonists [MRAs]) were not associated with an increased risk of hyperkalemia compared with placebo (hazard ratio (HR), 0.63 (95% CI, 0.48–0.83), *p*-heterogeneity = 0.90) [58].

In DAPA-HF, in which 70.1% of patients with HFrEF received MRAs at baseline, the risks of mild hyperkalemia and moderate-to-severe hyperkalemia were reduced with dapagliflozin by 14% (HR, 0.86 (95% CI, 0.70–1.05)) and 50% (HR, 0.50 (95% CI, 0.29–0.85)), respectively, compared with placebo [71]. In DAPA-CKD, the risk of hyperkalemia was reduced by 13% with dapagliflozin compared with placebo in patients with CKD (HR, 0.87 (95% CI, 0.70–1.09)), irrespective of MRA use at baseline (MRAs prescribed: HR, 0.94 (95% CI, 0.41–2.20); MRAs not prescribed: HR, 0.87 (95% CI, 0.69–1.10), *p*-interaction = 0.96); however, the results were not statistically significant [72]. Similarly, a secondary analysis of EMPEROR-Reduced demonstrated a non-statistically significant reduction in the risk of severe hyperkalemia with empagliflozin compared with placebo in patients with HFrEF (HR, 0.70 (95% CI, 0.47–1.04)), irrespective of MRA use at baseline [73]. The consensus among the physicians was that hyperkalemia is not a concern with SGLT-2is, even when used with MRAs.

### 3.6. Hypoglycemia

Due to the insulin-independent mechanism of action of SGLT-2is, the risk of hypoglycemia with these agents is low in patients with T2D but may increase when SGLT-2is are used concomitantly with insulin or sulfonylureas (insulin secretagogues) [28,29]. SGLT-2is have been studied in combination with other glucose-lowering agents (e.g., dipeptidyl peptidase-4 inhibitors, glucagon-like peptide 1 agonists, or metformin), and no significant incidence of hypoglycemia was observed [39]. In CVOTs, the incidence of hypoglycemia was not increased with empagliflozin [11], canagliflozin [9], or dapagliflozin [10] compared with placebo, despite approximately half the patients receiving concomitant insulin [39]. In contrast, a pooled analysis of clinical trials of empagliflozin showed an increased hypoglycemia incidence with concomitant sulfonylurea therapy [74]. There is no evidence to suggest that patients without T2D are at risk of hypoglycemia with SGLT-2is. No patients without T2D experienced major hypoglycemia in DAPA-HF [75] or severe hypoglycemia in DAPA-CKD [21].

If a patient with T2D is considered to be at risk of hypoglycemia (e.g., glycated hemoglobin <7.0–8.0%, history of hypoglycemia, CKD, or advanced age), the dose of sulfonylurea could be reduced or even stopped, while the insulin dose could be reduced by 10–20% prior to SGLT-2i initiation, with the frequency of blood glucose monitoring increased [39,40,75].

### 3.7. Amputation

An increased risk of amputation, primarily at the toe or metatarsal level, was observed with canagliflozin vs. placebo in the CANVAS study in patients with T2D and established CVD or high CVD risk (HR, 1.97 (95% CI, 1.41–2.75)) [9]. Specifically, the risk of amputation appeared to be the highest in patients with a history of amputation or peripheral artery disease (PAD) and was not dose-dependent [9,12]. Following CANVAS, a boxed warning was added to the prescribing information for canagliflozin regarding the risk of lower extremity amputation in patients with established CVD or high CVD risk, particularly in those with an increased risk of amputation, including those with PAD, prior amputations, neuropathy, and diabetic foot ulcers [76]. However, the incidence of amputations was not increased with canagliflozin in patients with T2D and CKD (CREDENCE [22]) or in cardiovascular and renal outcomes trials of empagliflozin in patients with T2D and established CVD (EMPA-REG OUTCOME [77]) or dapagliflozin in patients with T2D and established CVD or high CVD risk (DECLARE–TIMI 58 [10]), patients with HFrEF with or without T2D (DAPA-HF [23]), or patients with CKD with or without T2D (DAPA-CKD [21]).

The low risk of amputation with canagliflozin in CREDENCE resulted in the removal of the boxed warning of this event from the canagliflozin prescribing information [78]. However, physicians should advise patients about proper foot care; monitor at-risk patients for new pain, skin ulceration, or infections during canagliflozin treatment; and discontinue canagliflozin if indicated [12,28,39,79]. A similar recommendation has been made for ertugliflozin due to a potential increased risk of amputation, based on the incidence of non-traumatic lower limb amputation in clinical trials with ertugliflozin [38].

## 4. Conclusions

Clinical trial evidence has demonstrated that SGLT-2is provide beneficial effects on CV and renal outcomes in patients with and without T2D. Irrespective of these benefits, some of the key barriers to prescribing among physicians are related to understanding the safety profile of this drug class. However, serious adverse events in SGLT-2i users are relatively rare. Although some populations may demonstrate increased risk of some adverse events, most are easily managed and can be mitigated with increased physician education. By addressing SGLT-2i safety and increasing awareness of the cardiorenal effects, physicians will more readily integrate SGLT-2is into clinical practice as an option for patients who are likely to benefit. The use of SGLT-2is in broader patient populations, including patients without T2D, is increasing in clinical practice. Consequently, additional real-world evidence of their efficacy and safety will become available in the near future, particularly for patient populations typically excluded from or underrepresented in clinical trials. This is likely to enable physicians to further optimize treatment selection and management for individual patients with indications for SGLT-2i treatment.

## Figures and Tables

**Table 1:** Summary of glycemic, cardiovascular, and renal end points in the SGLT-2i outcome trials.

Scheme 1	Population	Median Follow-Up	HbA1c Difference vs. Placebo, Mean (95% CI)	CV Outcomes, HR (95% CI)	Renal Outcomes, HR (95% CI)
**Canagliflozin**					
CANVAS [9]	Age ≥ 30 y with T2D and established CVD OR Age ≥ 50 y with T2D and ≥2 CVD risk factors (*n* = 10,142)	~126 wk	−0.58% (−0.61%, −0.56%)	MACE ^a^: 0.86 (0.75–0.97); *p* < 0.001 for noninferiority and *p* = 0.02 for superiority CV death or HHF: 0.78 (0.67–0.91) HHF: 0.67 (0.52–0.87) CV death: 0.87 (0.72–1.06)	Progression of albuminuria: 0.73 (0.67–0.79) 40% reduction in eGFR, RRT initiation, or death from renal causes: 0.60 (0.47–0.77)
CREDENCE [22]	Age ≥30 y with T2D and CKD ^b^ (*n* = 4401)	~2.6 y	−0.25% (−0.31%, −0.20%)	MACE ^a^: 0.80 (0.67–0.95); *p* = 0.01 CV death or HHF: 0.69 (0.57–0.83) *p* < 0.001 HHF: 0.61 (0.47–0.80) *p* < 0.001 CV death: 0.78 (0.61–1.00); *p* = 0.05	ESKD, doubling of sCr, or death from renal causes: 0.66 (0.53–0.81); *p* < 0.001 Doubling of sCr: 0.60 (0.48–0.76); *p* < 0.001 ESKD: 0.68 (0.54–0.86); *p* = 0.002
**Dapagliflozin**					
DECLARE–TIMI 58 [10]	Age ≥ 40 y with T2D and established CVD OR Age ≥ 55 y (men) or ≥60 y (women) with T2D and ≥1 CVD risk factor (*n* = 17,160)	4.2 y	−0.42% (−0.45%, −0.40%)	MACE ^a^: 0.93 (0.84–1.03); *p* < 0.001 for noninferiority and *p* = 0.17 for superiority CV death or HHF: 0.83 (0.84–0.95); *p* = 0.005 HHF: 0.73 (0.61–0.88) CV death: 0.98 (0.82–1.17)	≥40% reduction in eGFR to <60 mL/min/1.73 m^2^, ESKD, or death from CV or renal causes: 0.76 (0.67–0.87) ≥40% reduction in eGFR to <60 mL/min/1.73 m^2^, ESKD, or death from renal causes: 0.53 (0.43–0.66)
DAPA-HF [23]	Age ≥18 y with NYHA class II–IV HFrEF (EF ≤40%) with or without T2D (*n* = 4744)	18.2 mo	−0.24% (−0.34%, −0.13%); *p* < 0.001 ^c^	Worsening HF ^d^ or CV death: 0.74 (0.65–0.85); *p* < 0.001 CV death or HHF: 0.75 (0.65–0.85); *p* < 0.001 Worsening HF ^d^: 0.70 (0.59–0.83) HHF: 0.70 (0.59–0.83) Urgent HF visit: 0.43 (0.20–0.90) CV death: 0.82 (0.69–0.98)	Worsening renal function ^e^: 0.71 (0.44–1.16)
DAPA-CKD [21]	Age ≥18 y with CKD ^f^ with or without T2D (*n* = 4094)	2.4 y	NR	CV death or HHF: 0.71 (0.55–0.92); *p* = 0.009 CV death: 0.81 (0.58–1.12)	Sustained ≥50% reduction in eGFR, ESKD, or death from CV or renal causes: 0.61 (0.51–0.72); *p* < 0.001 Sustained ≥50% reduction in eGFR, ESKD, or death from renal causes: 0.56 (0.45–0.68); *p* < 0.001 ≥ 50% reduction in eGFR: 0.53 (0.42–0.67) ESKD: 0.64 (0.50–0.82)
**Empagliflozin**					
EMPA-REG OUTCOME [11,24]	Age ≥18 y with T2D and established CVD (*n* = 7020)	3.1 y	Adjusted mean difference, 10 mg dose: −0.24% (−0.40%, −0.08%); 25 mg dose: −0.36% (−0.51%, −0.20%)	MACE ^a^: 0.86 (0.74–0.99); *p* < 0.001 for noninferiority and *p* = 0.04 for superiority MACE ^a^ or hospitalization for UA: 0.89 (0.78–1.01); *p* < 0.001 for noninferiority and *p* = 0.08 for superiority CV death or HHF: 0.66 (0.55–0.79); *p* < 0.001 HHF: 0.65 (0.50–0.85); *p* = 0.002 CV death: 0.62 (0.49–0.77); *p* < 0.001	Incident or worsening nephropathy ^g^: 0.61 (0.53–0.70); *p* < 0.001 Doubling of sCr with eGFR ≤45 mL/min/1.73 m^2^, RRT initiation, or death from renal causes: 0.54 (0.40–0.75); *p* < 0.001
EMPEROR-Reduced [25]	Age ≥18 y with NYHA class II–IV HFrEF (EF ≤40%) with or without T2D (*n* = 3730)	16 mo	Absolute difference: −0.16 (−0.25, −0.08) ^c^	CV death or HHF: 0.75 (0.65–0.86); *p* < 0.001 HHF: 0.69 (0.59–0.81) CV death: 0.92 (0.75–1.12)	Composite renal outcome ^h^: 0.50 (0.32–0.77)
EMPEROR-Preserved [26]	Age ≥18 y with NYHA class II–IV HFpEF (EF >40%) with or without T2D (*n* = 5988)	26.2 mo	Adjusted mean difference: −0.19% (−0.25%, −0.14%)^c^	CV death or HHF: 0.79 (0.69–0.90); *p* < 0.001 HHF: 0.71 (0.60–0.83) CV death: 0.91 (0.76–1.09)	Mean difference (95% CI) in eGFR slope change per year vs. placebo: 1.36 (1.06–1.66) mL/min/1.73 m^2^; *p* < 0.001 Composite renal outcome ^h^: 0.95 (0.73–1.24)
**Ertugliflozin**					
VERTIS CV [8]	Age ≥40 y with T2D and established CVD (*n* = 8246)	3.0 y	LSM difference at wk 18 vs. baseline, 5 mg: −0.70% (−0.73%, −0.67%); 15 mg: −0.72% (−0.75%, −0.69%); placebo: −0.22% (−0.25%, −0.19%)	MACE: 0.97 (0.85–1.11); *p* < 0.001 for noninferiority CV death or HHF: 0.88 (0.75–1.03); *p* = 0.11 HHF: 0.70 (0.54–0.90) CV death: 0.92 (0.77–1.11)	Doubling of sCr, RRT initiation, or death from renal causes: 0.81 (0.63–1.04)

^a^ Defined as the composite outcome of CV death, nonfatal myocardial infarction, or nonfatal stroke. ^b^ Defined as an eGFR of 30 –< 90 mL/min/1.73 m^2^ and a UACR of >300–5000 mg/g. ^c^ In patients with diabetes. ^d^ Defined as hospitalization or an urgent visit resulting in intravenous therapy for HF. ^e^ Defined as the composite outcome of ≥50% reduction in eGFR sustained for ≥28 days, ESKD, or death from renal causes. ^f^ Defined as an eGFR of 25–75 mL/min/1.73 m^2^ and a UACR of 200–5000 mg/g. ^g^ Defined as the composite outcome of progression to macroalbuminuria (UACR >300 mg/g), doubling of sCr accompanied by an eGFR of ≤45 mL/min/1.73 m^2^, initiation of RRT, or death from renal causes. ^h^ Defined as long-term dialysis or renal transplantation, a sustained reduction of ≥40% in eGFR, or a sustained eGFR of <15 mL/min/1.73 m^2^ (in those with baseline eGFR of ≥30 mL/min/1.73 m^2^) or <10 mL/min/1.73 m^2^ (in those with baseline eGFR of <30 mL/min/1.73 m^2^). CI, confidence interval; CKD, chronic kidney disease; CV, cardiovascular; CVD, cardiovascular disease; eGFR, estimated glomerular filtration rate; EF, ejection fraction; ESKD, end-stage kidney disease; HbA1c, glycated hemoglobin; HF, heart failure; HFpEF, HF with preserved ejection fraction; HFrEF, HF with reduced ejection fraction; HHF, hospitalization for HF; HR, hazard ratio; LSM, least squares mean; MACE, major adverse cardiovascular events; NR, not reported; NYHA, New York Heart Association; RRT, renal replacement therapy; sCr, serum creatinine; SGLT-2i, sodium-glucose cotransporter-2 inhibitor; T2D, type 2 diabetes; UA, unstable angina; UACR, urinary albumin-to-creatinine ratio.

**Table 2:** Guidelines for the use of SGLT-2is.

Year	Guidelines	SGLT-2is	Indications
2019	American College of Cardiology (ACC)/American Heart Association (AHA) [33]	Canagliflozin, dapagliflozin, and empagliflozin	T2D and ASCVD
2019	European Society of Cardiology (ESC) [34]	Canagliflozin, dapagliflozin, and empagliflozin	T2D and CVD
2020	Kidney Disease: Improving Global Outcomes (KDIGO) Clinical Practice Guideline for Diabetes Management in Chronic Kidney Disease [35]	Canagliflozin, dapagliflozin, and empagliflozin	T2D and CKD
2021	ESC/Heart Failure Association (HFA) of the ESC [37]	Canagliflozin, dapagliflozin, empagliflozin, ertugliflozin, and sotagliflozin	T2D and CVD
		Dapagliflozin, empagliflozin, and sotagliflozin	T2D and HFrEF
2021	ACC Expert Consensus Decision Pathway for Optimization of Heart Failure Treatment [36]	Dapagliflozin and ^a^ empagliflozin ^a^	HFrEF with or without T2D
2022	American Diabetes Association [31,32]	SGLT-2i drug class recommended An SGLT-2i with proven benefit for the individual patient’s comorbidities is recommended (CVD: canagliflozin, dapagliflozin, empagliflozin, and ertugliflozin; DKD: canagliflozin, dapagliflozin, and empagliflozin)	T2D, ASCVD, HF, and DKD

^a^ Prescribed in conjunction with a background of guideline-directed medical therapy for HF. ASCVD, atherosclerotic cardiovascular disease; CKD, chronic kidney disease; CVD, cardiovascular disease; DKD, diabetic kidney disease; HF, heart failure; HFrEF, HF with reduced ejection fraction; SGLT-2i, sodium-glucose cotransporter-2 inhibitor; T2D, type 2 diabetes.

**Table 3:** Summary of safety results from the SGLT-2i outcome trials.

Study Name	Population	Risk of AEs
**Canagliflozin**		
CANVAS [9]	Age ≥30 y with T2D and established CVD OR Age ≥50 y with T2D and ≥2 CVD risk factors (*n* = 10,142)	Any SAE (vs. placebo): HR, 0.93 (95% CI, 0.87–1.00); *p* = 0.04 AE leading to discontinuation (vs. placebo): HR, 1.13 (95% CI, 0.99–1.28); *p* = 0.07 Higher rate vs. placebo of amputation (*p* < 0.001), adjudicated fracture (*p* = 0.02), infection of male genitalia (*p* < 0.001), osmotic diuresis (*p* < 0.001), volume depletion (*p* = 0.009), and mycotic genital infection in women (*p* < 0.001)
CREDENCE [22]	Age ≥30 y with T2D and CKD ^a^ (*n* = 4401)	Any AE (vs. placebo): HR, 0.87 (95% CI, 0.82–0.93) Any SAE (vs. placebo): HR, 0.87 (95% CI, 0.79–0.97) Treatment-related SAEs (vs. placebo): HR, 1.45 (95% CI, 0.98–2.14) Higher risk vs. placebo of diabetic ketoacidosis (HR, 10.80 (95% CI, 1.39–83.65)) Similar risk vs. placebo of lower extremity amputation (HR, 1.11 (95% CI, 0.79–1.56)) and fracture (HR, 0.98 (95% CI, 0.70–1.37))
**Dapagliflozin**		
DECLARE–TIMI 58 [10]	Age ≥40 y with T2D and established CVD OR Age ≥55 y (men) or ≥60 y (women) with T2D and ≥1 CVD risk factor (*n* = 17,160)	Any SAE (vs. placebo): HR, 0.91 (95% CI, 0.87–0.96); *p* < 0.001 AE leading to discontinuation (vs. placebo): HR, 1.15 (95% CI, 1.03–1.28); *p* = 0.01 Lower risk vs. placebo of major hypoglycemic event (HR, 0.68; *p* = 0.02) and AKI (HR, 0.69; *p* = 0.002) Higher risk vs. placebo of diabetic ketoacidosis (HR, 2.18; *p* = 0.02) and genital infection (HR, 8.36; *p* < 0.001) Similar risk vs. placebo of amputation (HR, 1.09; *p* = 0.53), fracture (HR, 1.04; *p* = 0.59), volume depletion symptoms (HR, 1.00; *p* = 0.99), and UTI (HR, 0.93; *p* = 0.54)
DAPA-HF [23]	Age ≥18 y with NYHA class II–IV HFrEF (EF ≤ 40%) with or without T2D (*n* = 4744)	Any SAE (vs. placebo): 37.8% vs. 42.0% AE leading to discontinuation (vs. placebo): 4.7% vs. 4.9%; *p* = 0.79 Lower incidence vs. placebo of serious renal events (1.6% vs. 2.7%; *p* = 0.009) Similar incidence vs. placebo of SAEs related to volume depletion (1.2% vs. 1.7%; *p* = 0.23)
DAPA-CKD [21]	Age ≥18 y with CKD ^b^ with or without T2D (*n* = 4094)	Any SAE (vs. placebo): 29.5% vs. 33.9%; *p* = 0.002 AE leading to discontinuation (vs. placebo): 5.5% vs. 5.7%; *p* = 0.79 Lower incidence vs. placebo of major hypoglycemia (0.7% vs. 1.3%; *p* = 0.04) Higher incidence vs. placebo of volume depletion (5.9% vs. 4.2%; *p* = 0.01) Similar incidence vs. placebo of amputation (1.6% vs. 1.8%; *p* = 0.73), definite or probable diabetic ketoacidosis (0% vs. <0.1%; *p* = 0.50), fracture (4.0% vs. 3.2%; *p* = 0.22), and renal-related AE (7.2% vs. 8.7%; *p* = 0.07)
**Empagliflozin**		
EMPA-REG OUTCOME [11]	Age ≥18 y with T2D and established CVD (*n* = 7020)	Any SAE (vs. placebo): 38.2% vs. 42.3%; *p* < 0.001 AE leading to discontinuation (vs. placebo): 17.3% vs. 19.4%; *p* < 0.01 Lower incidence vs. placebo of UTI in women (36.4% vs. 40.6%; *p* < 0.05), AKI (1.0% vs. 1.6%; *p* < 0.05), and acute renal failure (5.2% vs. 6.6%; *p* < 0.01) Higher incidence vs. placebo of genital infection in men (5.0% vs. 1.5%; *p* < 0.001) and women (10.0% vs. 2.6%; *p* < 0.001) Similar incidence vs. placebo of hypoglycemia requiring assistance (1.3% vs. 1.5%), UTI (18.0% vs. 18.1%), complicated UTI (1.7% vs. 1.8%), volume depletion (5.1% vs. 4.9%), fracture (3.8% vs. 3.9%), thromboembolic event (0.6% vs. 0.9%), and diabetic ketoacidosis (0.1% vs. <0.1%)
EMPEROR-Reduced [25]	Age ≥18 y with NYHA class II–IV HFrEF (EF ≤40%) with or without T2D (*n* = 3730)	Any SAE (vs. placebo): 41.4% vs. 48.1% Higher incidence vs. placebo of genital infections (1.7% vs. 0.6%) Similar incidence vs. placebo of volume depletion (10.6% vs. 9.9%), hypotension (9.4% vs. 8.7%), symptomatic hypotension (5.7% vs. 5.5%), UTI (4.9% vs. 4.5%), fracture (2.4% vs. 2.3%), hypoglycemic event (1.4% vs. 1.5%), complicated UTI (1.0% vs. 0.8%), lower extremity amputation (0.7% vs. 0.5%), complicated genital infection (0.3% vs. 0.3%), and ketoacidosis (0% vs. 0%)
EMPEROR-Preserved [26]	Age ≥18 y with NYHA class II–IV HFpEF (EF >40%) with or without T2D (*n* = 5988)	Any SAE (vs. placebo): 47.9% vs. 51.6% AE leading to discontinuation (vs. placebo): 19.1% vs. 18.4% Higher incidence vs. placebo of hypotension (10.4% vs. 8.6%), UTI (9.9% vs. 8.1%), and genital infection (2.2% vs. 0.7%) Similar incidence vs. placebo of acute renal failure (12.1% vs. 12.8%), symptomatic hypotension (6.6% vs. 5.2%), fracture (4.5% vs. 4.2%), hypoglycemic event (2.4% vs. 2.6%), ketoacidosis (0.1% vs. 0.2%), complicated UTI (1.9% vs. 1.5%), lower extremity amputation (0.5% vs. 0.8%), and complicated genital infection (0.3% vs. 0.3%)
**Ertugliflozin**		
VERTIS CV [8]	Age ≥40 y with T2D and established CVD (*n* = 8246)	Any SAE (vs. placebo): risk difference, 1.2 (95% CI, −3.7, 1.4) (5 mg); −2.0 (95% CI, −4.5, 0.6) (15 mg) AE leading to discontinuation (vs. placebo): risk difference, 0.7 (95% CI, −0.7, 2.1) (5 mg); 0.5 (95% CI −0.9, 1.8) (15 mg) Higher risk vs. placebo of UTI (risk difference, 2.1 (*p* = 0.02; 5 mg); 1.8 (*p* = 0.03; 15 mg)), genital mycotic infection in women (risk difference, 3.6 (*p* < 0.001; 5 mg); 5.4 (*p* < 0.001; 15 mg)) and men (risk difference, 3.3 (*p* < 0.001; 5 mg); 4.0 (*p* < 0.001; 15 mg)) Similar risk vs. placebo of symptomatic hypoglycemia (risk difference, −0.8 (5 mg); −2.3 (15 mg)), severe hypoglycemia (risk difference, −0.9 (5 mg); −0.5 (15 mg)), hypovolemia (risk difference, 0.4 (5 mg); 0.4 (15 mg)), AKI (risk difference −0.4 (5 mg); −0.3 (15 mg)), and amputation (pooled risk difference, 0.1) Similar incidence vs. placebo of fractures (3.6% (5 mg) and 3.7% (15 mg) vs. 3.6%), serious AKI (0.9% (5 mg) and 0.7% (15 mg) vs. 0.8%), and serious UTI (0.9% (5 mg) and 0.4% (15 mg) vs. 0.8%)

^a^ Defined as an eGFR of 30 –< 90 mL/min/1.73 m^2^ and a UACR of >300–5000 mg/g. ^b^ Defined as an eGFR of 25–75 mL/min/1.73 m^2^ and a UACR of 200–5000 mg/g. AE, adverse event; AKI, acute kidney injury; CI, confidence interval; CKD, chronic kidney disease; CVD, cardiovascular disease; eGFR, estimated glomerular filtration rate; EF, ejection fraction; HF, heart failure; HFpEF, HF with preserved ejection fraction; HFrEF, HF with reduced ejection fraction; HR, hazard ratio; NYHA, New York Heart Association; SAE, serious AE; SGLT-2i, sodium-glucose cotransporter-2 inhibitor; T2D, type 2 diabetes; UACR, urinary albumin-to-creatinine ratio; UTI, urinary tract infection.

## Supplementary Materials

The following supporting information can be downloaded at: https://www.mdpi.com/article/10.3390/jcm11206051/s1. Figure S1: Chemical structure of sodium-glucose cotransporter-2 inhibitors approved for use in the United States [27,28,29,38].

## References

[1] Johansen M.E., Argyropoulos C. (2020). The cardiovascular outcomes, heart failure and kidney disease trials tell that the time to use sodium glucose cotransporter 2 inhibitors is now. Clin. Cardiol..

[2] Neuen B.L., Cherney D.Z., Jardine M.J., Perkovic V. (2019). Sodium-glucose cotransporter inhibitors in type 2 diabetes: Thinking beyond glucose lowering. CMAJ.

[3] Vaduganathan M., Sathiyakumar V., Singh A., McCarthy C.P., Qamar A., Januzzi J.L., Scirica B.M., Butler J., Cannon C.P., Bhatt D.L. (2018). Prescriber patterns of SGLT2i after expansions of U.S. Food and Drug Administration labeling. J. Am. Coll. Cardiol..

[4] Rieg T., Vallon V. (2018). Development of SGLT1 and SGLT2 inhibitors. Diabetologia.

[5] Seufert J. (2015). SGLT2 inhibitors—An insulin-independent therapeutic approach for treatment of type 2 diabetes: Focus on canagliflozin. Diabetes Metab. Syndr. Obes..

[6] Piperidou A., Loutradis C., Sarafidis P. (2021). SGLT-2 inhibitors and nephroprotection: Current evidence and future perspectives. J. Hum. Hypertens..

[7] US Food and Drug Administration Guidance for Industry: Diabetes Mellitus—Evaluating Cardiovascular Risk in New Antidiabetic Therapies to Treat Type 2 Diabetes. https://www.fda.gov/media/71297/download.

[8] Cannon C.P., Pratley R., Dagogo-Jack S., Mancuso J., Huyck S., Masiukiewicz U., Charbonnel B., Frederich R., Gallo S., Cosentino F. (2020). Cardiovascular outcomes with ertugliflozin in type 2 diabetes. N. Engl. J. Med..

[9] Neal B., Perkovic V., Mahaffey K.W., de Zeeuw D., Fulcher G., Erondu N., Shaw W., Law G., Desai M., Matthews D.R. (2017). Canagliflozin and cardiovascular and renal events in type 2 diabetes. N. Engl. J. Med..

[10] Wiviott S.D., Raz I., Bonaca M.P., Mosenzon O., Kato E.T., Cahn A., Silverman M.G., Zelniker T.A., Kuder J.F., Murphy S.A. (2019). Dapagliflozin and cardiovascular outcomes in type 2 diabetes. N. Engl. J. Med..

[11] Zinman B., Wanner C., Lachin J.M., Fitchett D., Bluhmki E., Hantel S., Mattheus M., Devins T., Johansen O.E., Woerle H.J. (2015). Empagliflozin, cardiovascular outcomes, and mortality in type 2 diabetes. N. Engl. J. Med..

[12] Vardeny O., Vaduganathan M. (2019). Practical guide to prescribing sodium-glucose cotransporter 2 inhibitors for cardiologists. JACC Heart Fail..

[13] Lupsa B.C., Inzucchi S.E. (2018). Use of SGLT2 inhibitors in type 2 diabetes: Weighing the risks and benefits. Diabetologia.

[14] DeFronzo R.A., Norton L., Abdul-Ghani M. (2017). Renal, metabolic and cardiovascular considerations of SGLT2 inhibition. Nat. Rev. Nephrol..

[15] O’Meara E., McDonald M., Chan M., Ducharme A., Ezekowitz J.A., Giannetti N., Grzeslo A., Heckman G.A., Howlett J.G., Koshman S.L. (2020). CCS/CHFS heart failure guidelines: Clinical trial update on functional mitral regurgitation, SGLT2 inhibitors, ARNI in HFpEF, and tafamidis in amyloidosis. Can. J. Cardiol..

[16] Staels B. (2017). Cardiovascular protection by sodium glucose cotransporter 2 inhibitors: Potential mechanisms. Am. J. Med..

[17] Vallon V., Thomson S.C. (2017). Targeting renal glucose reabsorption to treat hyperglycaemia: The pleiotropic effects of SGLT2 inhibition. Diabetologia.

[18] Lytvyn Y., Bjornstad P., Udell J.A., Lovshin J.A., Cherney D.Z.I. (2017). Sodium glucose cotransporter-2 inhibition in heart failure: Potential mechanisms, clinical applications, and summary of clinical trials. Circulation.

[19] Joshi S.S., Singh T., Newby D.E., Singh J. (2021). Sodium-glucose co-transporter 2 inhibitor therapy: Mechanisms of action in heart failure. Heart.

[20] Verma S., McMurray J.J.V. (2019). The serendipitous story of SGLT2 inhibitors in heart failure. Circulation.

[21] Heerspink H.J.L., Stefánsson B.V., Correa-Rotter R., Chertow G.M., Greene T., Hou F.F., Mann J.F.E., McMurray J.J.V., Lindberg M., Rossing P. (2020). Dapagliflozin in patients with chronic kidney disease. N. Engl. J. Med..

[22] Perkovic V., Jardine M.J., Neal B., Bompoint S., Heerspink H.J.L., Charytan D.M., Edwards R., Agarwal R., Bakris G., Bull S. (2019). Canagliflozin and renal outcomes in type 2 diabetes and nephropathy. N. Engl. J. Med..

[23] McMurray J.J.V., Solomon S.D., Inzucchi S.E., Køber L., Kosiborod M.N., Martinez F.A., Ponikowski P., Sabatine M.S., Anand I.S., Bělohlávek J. (2019). Dapagliflozin in patients with heart failure and reduced ejection fraction. N. Engl. J. Med..

[24] Wanner C., Inzucchi S.E., Lachin J.M., Fitchett D., von Eynatten M., Mattheus M., Johansen O.E., Woerle H.J., Broedl U.C., Zinman B. (2016). Empagliflozin and progression of kidney disease in type 2 diabetes. N. Engl. J. Med..

[25] Packer M., Anker S.D., Butler J., Filippatos G., Pocock S.J., Carson P., Januzzi J., Verma S., Tsutsui H., Brueckmann M. (2020). Cardiovascular and renal outcomes with empagliflozin in heart failure. N. Engl. J. Med..

[26] Anker S.D., Butler J., Filippatos G., Ferreira J.P., Bocchi E., Böhm M., Brunner-La Rocca H.P., Choi D.J., Chopra V., Chuquiure-Valenzuela E. (2021). Empagliflozin in heart failure with a preserved ejection fraction. N. Engl. J. Med..

[27] Boehringer Ingelheim Jardiance® (Empagliflozin Tablets), for Oral Use [Prescribing Information]. https://docs.boehringer-ingelheim.com/Prescribing%20Information/PIs/Jardiance/jardiance.pdf.

[28] Janssen Pharmaceuticals Invokana® (Canagliflozin) Tablets, for Oral Use [Prescribing Information]. https://www.janssenlabels.com/package-insert/product-monograph/prescribing-information/INVOKANA-pi.pdf.

[29] AstraZeneca Farxiga® (Empagliflozin) Tablets, for Oral Use [Prescribing Information]. https://den8dhaj6zs0e.cloudfront.net/50fd68b9-106b-4550-b5d0-12b045f8b184/0be9cb1b-3b33-41c7-bfc2-04c9f718e442/0be9cb1b-3b33-41c7-bfc2-04c9f718e442_viewable_rendition__v.pdf.

[30] American Diabetes Association Professional Practice Committee (2022). 11. Chronic kidney disease and risk management: Standards of Medical Care in Diabetes—2022. Diabetes Care.

[31] American Diabetes Association Professional Practice Committee (2022). 10. Cardiovascular disease and risk management: Standards of Medical Care in Diabetes—2022. Diabetes Care.

[32] American Diabetes Association Professional Practice Committee (2022). 9. Pharmacologic approaches to glycemic treatment: Standards of Medical Care in Diabetes—2022. Diabetes Care.

[33] Arnett D.K., Blumenthal R.S., Albert M.A., Buroker A.B., Goldberger Z.D., Hahn E.J., Himmelfarb C.D., Khera A., Lloyd-Jones D., McEvoy J.W. (2019). 2019 ACC/AHA guideline on the primary prevention of cardiovascular disease. J. Am. Coll. Cardiol..

[34] Cosentino F., Grant P.J., Aboyans V., Bailey C.J., Ceriello A., Delgado V., Federici M., Filippatos G., Grobbee D.E., Hansen T.B. (2020). 2019 ESC guidelines on diabetes, pre-diabetes, and cardiovascular diseases developed in collaboration with the EASD. Eur. Heart J..

[35] De Boer I.H., Caramori M.L., Chan J.C.N., Heerspink H.J.L., Hurst C., Khunti K., Liew A., Michos E.D., Navaneethan S.D., Olowu W.A. (2020). KDIGO 2020 clinical practice guideline for diabetes management in chronic kidney disease. Kidney Int..

[36] Maddox T.M., Januzzi J.L., Allen L.A., Breathett K., Butler J., Davis L.L., Fonarow G.C., Ibrahim N.E., Lindenfeld J., Masoudi F.A. (2021). 2021 update to the 2017 ACC expert consensus decision pathway for optimization of heart failure treatment: Answers to 10 pivotal issues about heart failure with reduced ejection fraction: A report of the American College of Cardiology Solution Set Oversight Committee. J. Am. Coll. Cardiol..

[37] McDonagh T.A., Metra M., Adamo M., Gardner R.S., Baumbach A., Böhm M., Burri H., Butler J., Čelutkienė J., Chioncel O. (2021). 2021 ESC guidelines for the diagnosis and treatment of acute and chronic heart failure. Eur. Heart J..

[38] Merck & Co Steglatro® (Ertugliflozin) Tablets, for Oral Use [Prescribing Information]. https://www.merck.com/product/usa/pi_circulars/s/steglatro/steglatro_pi.pdf.

[39] Fitchett D. (2019). A safety update on sodium glucose co-transporter 2 inhibitors. Diabetes Obes. Metab..

[40] Das S.R., Everett B.M., Birtcher K.K., Brown J.M., Januzzi J.L., Kalyani R.R., Kosiborod M., Magwire M., Morris P.B., Neumiller J.J. (2020). 2020 Expert consensus decision pathway on novel therapies for cardiovascular risk reduction in patients with type 2 diabetes: A report of the American College of Cardiology Solution Set Oversight Committee. J. Am. Coll. Cardiol..

[41] Van Bommel E.J.M., Muskiet M.H.A., van Baar M.J.B., Tonneijck L., Smits M.M., Emanuel A.L., Bozovic A., Danser A.H.J., Geurts F., Hoorn E.J. (2020). The renal hemodynamic effects of the SGLT2 inhibitor dapagliflozin are caused by post-glomerular vasodilatation rather than pre-glomerular vasoconstriction in metformin-treated patients with type 2 diabetes in the randomized, double-blind RED trial. Kidney Int..

[42] Kraus B.J., Weir M.R., Bakris G.L., Mattheus M., Cherney D.Z.I., Sattar N., Heerspink H.J.L., Ritter I., von Eynatten M., Zinman B. (2021). Characterization and implications of the initial estimated glomerular filtration rate ‘dip’ upon sodium-glucose cotransporter-2 inhibition with empagliflozin in the EMPA-REG OUTCOME trial. Kidney Int..

[43] Heerspink H.J.L., Cherney D.Z.I. (2021). Clinical implications of an acute dip in eGFR after SGLT2 inhibitor initiation. Clin. J. Am. Soc. Nephrol..

[44] Meraz-Muñoz A.Y., Weinstein J., Wald R. (2021). eGFR decline after SGLT2 inhibitor initiation: The tortoise and the hare reimagined. Kidney360.

[45] Chertow G.M., Vart P., Jongs N., Toto R.D., Gorriz J.L., Hou F.F., McMurray J.J.V., Correa-Rotter R., Rossing P., Sjöström C.D. (2021). Effects of dapagliflozin in stage 4 chronic kidney disease. J. Am. Soc. Nephrol..

[46] Iskander C., Cherney D.Z., Clemens K.K., Dixon S.N., Harel Z., Jeyakumar N., McArthur E., Muanda F.T., Parikh C.R., Paterson J.M. (2020). Use of sodium-glucose cotransporter-2 inhibitors and risk of acute kidney injury in older adults with diabetes: A population-based cohort study. CMAJ.

[47] Nadkarni G.N., Ferrandino R., Chang A., Surapaneni A., Chauhan K., Poojary P., Saha A., Ferket B., Grams M.E., Coca S.G. (2017). Acute kidney injury in patients on SGLT2 inhibitors: A propensity-matched analysis. Diabetes Care.

[48] Rampersad C., Kraut E., Whitlock R.H., Komenda P., Woo V., Rigatto C., Tangri N. (2020). Acute kidney injury events in patients with type 2 diabetes using SGLT2 inhibitors versus other glucose-lowering drugs: A retrospective cohort study. Am. J. Kidney Dis..

[49] Xie Y., Bowe B., Gibson A.K., McGill J.B., Maddukuri G., Al-Aly Z. (2021). Clinical implications of estimated glomerular filtration rate dip following sodium-glucose cotransporter-2 inhibitor initiation on cardiovascular and kidney outcomes. J. Am. Heart Assoc..

[50] Zhao M., Sun S., Huang Z., Wang T., Tang H. (2020). Network meta-analysis of novel glucose-lowering drugs on risk of acute kidney injury. Clin. J. Am. Soc. Nephrol..

[51] Wilcox C.S. (2020). Antihypertensive and renal mechanisms of SGLT2 (sodium-glucose linked transporter 2) inhibitors. Hypertension.

[52] Serenelli M., Böhm M., Inzucchi S.E., Køber L., Kosiborod M.N., Martinez F.A., Ponikowski P., Sabatine M.S., Solomon S.D., DeMets D.L. (2020). Effect of dapagliflozin according to baseline systolic blood pressure in the Dapagliflozin and Prevention of Adverse Outcomes in Heart Failure trial (DAPA-HF). Eur. Heart J..

[53] Seferovic P.M., Ponikowski P., Anker S.D., Bauersachs J., Chioncel O., Cleland J.G.F., de Boer R.A., Drexel H., Ben Gal T., Hill L. (2019). Clinical practice update on heart failure 2019: Pharmacotherapy, procedures, devices and patient management. An expert consensus meeting report of the Heart Failure Association of the European Society of Cardiology. Eur. J. Heart Fail..

[54] Rong X., Li X., Gou Q., Liu K., Chen X. (2020). Risk of orthostatic hypotension associated with sodium-glucose cotransporter-2 inhibitor treatment: A meta-analysis of randomized controlled trials. Diabetes Vasc. Dis. Res..

[55] Cherney D.Z.I., Udell J.A. (2016). Use of sodium glucose cotransporter 2 inhibitors in the hands of cardiologists. Circulation.

[56] Mistry S., Eschler D.C. (2021). Euglycemic diabetic ketoacidosis caused by SGLT2 inhibitors and a ketogenic diet: A case series and review of literature. AACE Clin. Case Rep..

[57] Goldenberg R.M., Berard L.D., Cheng A.Y.Y., Gilbert J.D., Verma S., Woo V.C., Yale J.-F. (2016). SGLT2 inhibitor–associated diabetic ketoacidosis: Clinical review and recommendations for prevention and diagnosis. Clin. Ther..

[58] Toyama T., Neuen B.L., Jun M., Ohkuma T., Neal B., Jardine M.J., Heerspink H.L., Wong M.G., Ninomiya T., Wada T. (2019). Effect of SGLT2 inhibitors on cardiovascular, renal and safety outcomes in patients with type 2 diabetes mellitus and chronic kidney disease: A systematic review and meta-analysis. Diabetes Obes. Metab..

[59] Palmer B.F., Clegg D.J., Taylor S.I., Weir M.R. (2016). Diabetic ketoacidosis, sodium glucose transporter-2 inhibitors and the kidney. J. Diabetes Complicat..

[60] Peters A.L., Buschur E.O., Buse J.B., Cohan P., Diner J.C., Hirsch I.B. (2015). Euglycemic diabetic ketoacidosis: A potential complication of treatment with sodium-glucose cotransporter 2 inhibition. Diabetes Care.

[61] Nyirjesy P., Sobel J.D. (2013). Genital mycotic infections in patients with diabetes. Postgrad. Med..

[62] McGovern A.P., Hogg M., Shields B.M., Sattar N.A., Holman R.R., Pearson E.R., Hattersley A.T., Jones A.G., Dennis J.M. (2020). Risk factors for genital infections in people initiating SGLT2 inhibitors and their impact on discontinuation. BMJ Open Diabetes Res. Care.

[63] Engelhardt K., Ferguson M., Rosselli J.L. (2021). Prevention and management of genital mycotic infections in the setting of sodium-glucose cotransporter 2 inhibitors. Ann. Pharmacother..

[64] Williams S.M., Ahmed S.H. (2019). Improving compliance with SGLT2 inhibitors by reducing the risk of genital mycotic infections: The outcomes of personal hygiene advice. Diabetes.

[65] Centers for Disease Control and Prevention (CDC) Vaginal Candidiasis. https://www.cdc.gov/fungal/diseases/candidiasis/genital/index.html.

[66] Petruski-Ivleva N., Schneeweiss S., Eapen S., Rajan A., Jan S. (2020). Fournier’s gangrene in patients with type 2 diabetes using second-line antidiabetic medications. Diabetes Obes. Metab..

[67] Tran B.A., Updike W.H., Bullers K., Serag-Bolos E. (2022). Sodium–glucose cotransporter 2 inhibitor use associated with Fournier’s gangrene: A review of case reports and spontaneous post-marketing cases. Clin. Diabetes.

[68] US Food and Drug Administration FDA Warns about Rare Occurrences of a Serious Infection of the Genital Area with SGLT2 Inhibitors for Diabetes. https://www.fda.gov/Drugs/DrugSafety/ucm617360.htm.

[69] Weir M.R., Kline I., Xie J., Edwards R., Usiskin K. (2014). Effect of canagliflozin on serum electrolytes in patients with type 2 diabetes in relation to estimated glomerular filtration rate (eGFR). Curr. Med. Res. Opin..

[70] Weir M.R., Slee A., Sun T., Balis D., Oh R., de Zeeuw D., Perkovic V. (2021). Effects of canagliflozin on serum potassium in the CANagliflozin cardioVascular Assessment Study (CANVAS) Program. Clin. Kidney J..

[71] Kristensen S.L., Docherty K.F., Jhund P.S., Bengtsson O., Demets D.L., Inzucchi S.E., Kober L., Kosiborod M.N., Langkilde A.M., Martinez F.A. (2020). Dapagliflozin reduces the risk of hyperkalaemia in patients with heart failure and reduced ejection fraction: A secondary analysis DAPA-HF. Eur. Heart J..

[72] Provenzano M., Jongs N., Vart P., Stefánsson B.V., Chertow G.M., Langkilde A.M., McMurray J.J.V., Correa-Rotter R., Rossing P., Sjöström C.D. (2021). The kidney protective effects of the sodium–glucose cotransporter-2 inhibitor, dapagliflozin, are present in patients with CKD treated with mineralocorticoid receptor antagonists. Kidney Int. Rep..

[73] Ferreira J.P., Zannad F., Pocock S.J., Anker S.D., Butler J., Filippatos G., Brueckmann M., Jamal W., Steubl D., Schueler E. (2021). Interplay of mineralocorticoid receptor antagonists and empagliflozin in heart failure: EMPEROR-Reduced. J. Am. Coll. Cardiol..

[74] Kohler S., Zeller C., Iliev H., Kaspers S. (2017). Safety and tolerability of empagliflozin in patients with type 2 diabetes: Pooled analysis of phase I-III clinical trials. Adv. Ther..

[75] Petrie M.C., Verma S., Docherty K.F., Inzucchi S.E., Anand I., Belohlávek J., Böhm M., Chiang C.E., Chopra V.K., de Boer R.A. (2020). Effect of dapagliflozin on worsening heart failure and cardiovascular death in patients with heart failure with and without diabetes. JAMA.

[76] US Food and Drug Administration Invokana (Canagliflozin) Tablets, for Oral Use [Prescribing Information]. http://www.accessdata.fda.gov/drugsatfda_docs/label/2017/204042s026lbl.pdf.

[77] Inzucchi S.E., Iliev H., Pfarr E., Zinman B. (2018). Empagliflozin and assessment of lower-limb amputations in the EMPA-REG OUTCOME trial. Diabetes Care.

[78] US Food and Drug Administration FDA Removes Boxed Warning about Risk of Leg and Foot Amputations for the Diabetes Medicine Canagliflozin (Invokana, Invokamet, Invokamet XR). https://www.fda.gov/drugs/drug-safety-and-availability/fda-removes-boxed-warning-about-risk-leg-and-foot-amputations-diabetes-medicine-canagliflozin.

[79] American Diabetes Association Professional Practice Committee (2022). 12. Retinopathy, neuropathy, and foot care: Standards of Medical Care in Diabetes—2022. Diabetes Care.

