# Supplementary material for: Physicians’ Considerations and Practice Recommendations Regarding the Use of Sodium-Glucose Cotransporter-2 Inhibitors
Source: J Clin Med. 2022 Oct 13;11(20):6051. doi: 10.3390/jcm11206051 (PMC9604628; doi:10.3390/jcm11206051)
Supplement: Supplementary file 1 [file jcm-11-06051-s001.zip › jcm-1850569-supplementary.pdf]

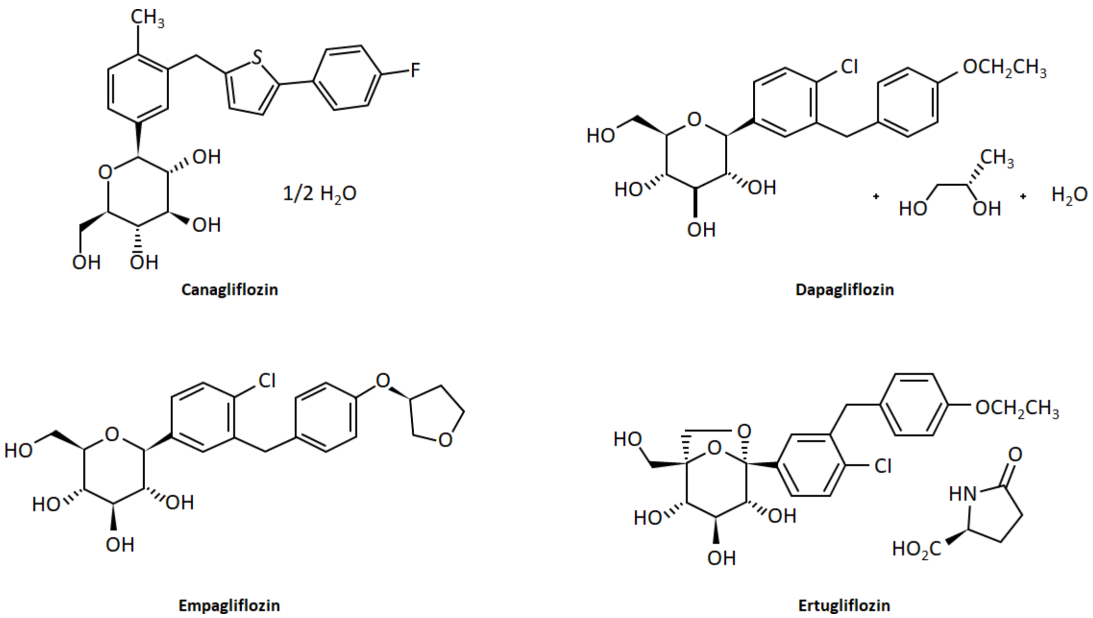

Supplementary Figure S1. Chemical structure of sodium-glucose cotransporter-2 inhibitors approved for use in the United States [27–29,38].
